# Sex differences in monitoring and achievement of blood pressure and LDL-cholesterol targets in chronic coronary syndrome

**DOI:** 10.48101/ujms.v131.14047

**Published:** 2026-08-31

**Authors:** Elin Täufer Cederlöf, Jessica Schubert, Nina Johnston, Maria K. Svensson, Thomas Cars, Lovisa Schalin, Emil Hagström

**Affiliations:** a Department of Medical Sciences, Cardiology and Department of Medical Sciences, Renal Medicine, Uppsala University, Uppsala, Sweden; b Renal Medicine, Department of Medical Sciences, Uppsala University, Uppsala, Sweden; c Uppsala Clinical Research Centre, Uppsala University, Uppsala, Sweden; d Sence Research AB, Uppsala, Sweden; e Amgen AB, Solna, Sweden

**Keywords:** Coronary artery disease, secondary prevention, LDL cholesterol, blood pressure, sex differences

## Abstract

**Background:**

The cornerstones of secondary prevention in chronic coronary syndrome (CCS) are blood pressure lowering and lipid-lowering therapy (LLT). The purpose of this study was to examine the control of systolic blood pressure (SBP) and low-density lipoprotein cholesterol (LDL-C) in patients with CCS followed in primary care.

**Methods:**

This was a retrospective population-based cohort study of patients diagnosed with CCS at cardiology clinics in Uppsala Region, Sweden, between 2012 and 2020. Data from electronic health records were analyzed for SBP and LDL-C monitoring and secondary prevention target achievement during 3 years of follow-up, stratified by sex, age, and diabetes status. Sex differences in time to LLT initiation during the first year after CCS diagnosis were evaluated using Cox proportional hazards models.

**Results:**

In total, 1,452 patients (415 women and 1,037 men) were followed for a median of 3.2 years (interquartile range [IQR]: 1.4–5.8). Most patients had at least one SBP measurement during follow-up, and approximately half met SBP targets (50.9% of women and 56.1% of men at year 1). LDL-C monitoring was less common: at year 1, 40.8% of women and 28.7% of men lacked a measurement, and targets were achieved in 12.1% of women and 24.7% of men. By year 3, target achievement had declined further to 11.2 and 15.0%, respectively. Women were less likely than men to initiate LLT within the first year (adjusted hazard ratio 0.70, 95% confidence interval: 0.61–0.80, *P* < 0.001). LLT use also declined over time, with 54.1% of women versus 73.9% of men on treatment at year 3 (*P* < 0.001).

**Conclusion:**

Large gaps in secondary prevention of patients with CCS were observed, with under-monitoring of LDL-C and declining use of LLT, particularly among women. Although SBP was routinely assessed, only half of patients achieved recommended levels. These findings highlight the need for systematic strategies in primary care to improve management in CCS.

## Introduction

Structured cardiac rehabilitation and evidence-based secondary prevention interventions have been shown to improve both short- and long-term prognoses in patients with coronary artery disease (1, 2). However, despite these interventions being widely available and relatively low in cost, most patients do not meet guideline-directed secondary prevention targets at the European level (3). Blood pressure lowering and lipid-lowering therapy (LLT) are cornerstones in secondary prevention, yet many patients still experience suboptimal management, with a substantial variability between centers in Europe (4). In Sweden, around half of the patients after a myocardial infarction (MI) reach blood pressure and cholesterol targets during the first year of follow-up (5, 6). While there are abundant data on treatment patterns and achievement for patients with MI, less is known about patients with chronic coronary syndrome (CCS), particularly among those managed in primary care (7). Although guidelines recommend the same blood pressure and low-density lipoprotein cholesterol (LDL-C) targets for women and men, recent data indicate that women generally have lower target achievement and a higher subsequent risk (8, 9). The underlying factors for women less often achieving targets include being undertreated, having different care-seeking patterns, and biological factors such as hormonal influences and pathophysiological mechanisms (10, 11). Notably, guideline-recommended LDL-C targets in patients with CCS are independent of age (12).

The aim of this study was to provide long-term data, beyond specialized care, on secondary preventive care in patients with CCS, and to describe the proportion of patients prescribed guideline-directed medical treatment over time after a diagnosis of CCS, as well as frequency of LDL-C and blood pressure monitoring, and target achievements by sex.

## Materials and methods

### Study setting

This population-based, retrospective, observational cohort study included patients in the County of Uppsala, Sweden, who received a first-time diagnosis of CCS at a cardiology clinic between January 1, 2012, and December 31, 2020. Data were obtained from tertiary (Uppsala University Hospital, Uppsala), secondary (Enköping Hospital, Enköping), and primary care centers in Region Uppsala, covering approximately 390,000 inhabitants (Supplementary material, Figure S1). Patients under 18 years of age and patients not resident in Uppsala County at time of diagnosis were excluded.

### Data sources

This study utilized data from Electronic Health Records (EHRs) from 2005 to 2020, including both inpatient care and outpatient visits at cardiology clinics as well as visits to both private and public primary care centers in the region. The dataset provided detailed information on patient demographics, diagnoses, clinical procedure codes, laboratory results, clinical measurements and drug utilization.

### Patients

All patients with a first-time diagnosis of CCS between 01 January 2012 and 31 December 2020 were included, and those with a prior diagnosis of acute coronary syndrome (ACS) were excluded. A patient was classified as having CCS based on a registered diagnosis of CCS, within 6 months following a procedure of either invasive or non-invasive coronary investigation or an invasive coronary intervention. The 6-month period was chosen to allow a coronary investigation to be performed and a subsequent referral and visit to a cardiology specialist to make a formal diagnosis. The date of first CCS diagnosis was designated as the index date and the start of the follow-up. All ICD-10 codes and procedure codes used to identify the study population are provided in the supplemental material, Table S1.

### Patient characteristics

Diagnoses (recorded in any position), clinical measurements, and laboratory results from primary care, specialized outpatient care, and inpatient care, both prior to and at the index date, were used to identify prevalent comorbid conditions. Definitions of comorbidities are described in the supplemental material (Supplemental Material, Table S1). Patients with diabetes were studied separately, since they have a more structured annual follow-up in primary care in Sweden, with visits and follow-up data registered in the Swedish National Diabetes Register (13, 14). The estimated glomerular filtration rate (eGFR) was calculated from plasma creatinine using the Lund–Malmö revised formula (15). To assess healthcare utilization and frailty, we used the Charlson Comorbidity Index (16), along with the total number of outpatient visits and inpatient episodes in the preceding year.

### Outcomes

The primary outcomes were number of assessments and target achievement of office-measured systolic blood pressure (SBP), LDL-C levels, and the dispensation of LLT. Prescription of LLT was defined as at least one filled prescription per calendar year from ATC class C10AA or C10AX09, based on the nationwide dispensation database integrated into the EHR, and this reflects medication access and continued treatment indication. The SBP target was defined as <140 mmHg. Since guideline-recommended LDL-C targets changed during the study period, we present target achievement against both thresholds (<1.8 and < 1.4 mmol/L). The use of prevalent LLT was defined as any dispensation of a statin and/or ezetimibe within 1 year before and up to 3 years after the index date.

### Follow-up

Patients were followed from the index date until the first occurrence of an ACS, emigration from Region Uppsala, death, or the end of the study (31 December 2020), whichever came first.

### Statistical analysis

Categorical variables were reported as frequencies and percentages, whereas continuous variables were presented as means with standard deviations (SDs) or medians with interquartile ranges (IQRs). SBP and LDL-C were analyzed for both frequency of assessment and target attainment at 1, 2, and 3 years post-index, restricted to patients with complete follow-up for each period. Additionally, a graphical assessment of SBP and LDL-C was conducted from 1 year prior to the index date through 3 years post-index, with each patient followed until their censoring date. Missing values were imputed from the mean value of the population (for eGFR and body mass index [BMI]). The frequency of missing values was 0.6% (*n* = 8) for eGFR and 13.7% (*n* = 199) for BMI. The E-value for the adjusted hazard ratio and the limit of the 95% confidence interval (CI) closest to the null were calculated. The E-value is defined as the minimum strength of association that one or more unmeasured confounders would need to have with both the exposure and the outcome to fully explain away a specific exposure-outcome association, conditional on the measured covariates (17).

Analyses were stratified by age (< 60, 60– < 70, 70– < 80, and ≥80 years), and subgroup analyses were performed separately for women and men and for patients with and without diabetes mellitus. To further investigate potential sex-based differences in LLT initiation within the first year following CCS diagnosis, a Cox proportional hazards model was used to estimate the time from CCS diagnosis to the first dispensation of LLT. Patients were followed for up to 1 year from CCS diagnosis, with censoring at death, recurrent ACS, or emigration from the region. The model was adjusted for age and eGFR (both modeled as restricted cubic splines with four knots), sex, hypertension, diabetes mellitus, prior ischemic stroke, peripheral artery disease, cancer, Charlson Comorbidity Index, and healthcare utilization (total number of outpatient visits, inpatient episodes, and medication dispensed in sachets in the preceding year).

For LLT, the proportion of patients classified as treated was estimated from 1 year before the first CCS diagnosis to 3 years after the index date. A patient was considered treated on any given day if they had medication coverage for that day, calculated as the total number of dispensed tablets plus an additional 25% to account for variations in adherence. In a sensitivity analysis, we examined the characteristics of patients who did not receive LLT within 1 year after the index date. This analysis was restricted to patients with at least 1 year of follow-up. We also examined factors associated with pharmacy dispensation of LLT within 1 year after the first CCS event among patients with at least 365 days of follow-up. A multivariable logistic regression model was fitted with LLT dispensation within 1 year as the dependent variable and age, sex, BMI, eGFR, hypertension, diabetes, renal failure, cancer, ischemic stroke, peripheral artery disease, number of outpatient visits, number of inpatient episodes, Charlson Comorbidity Index, number of unique dispensed drugs, medication dispensed in sachets, coronary angiography, percutaneous coronary intervention (PCI), and previous LLT use in the preceding year as independent variables. Differences in proportions of SBP monitoring, LDL-C monitoring, target achievement, and LLT use between women and men, and between patients with and without diabetes, were assessed using the Pearson’s chi-squared test. Changes in proportions between years 1 and 3 were assessed using the McNemar’s test, restricted to patients with at least 3 years of follow-up to allow paired comparisons within the same population. Data management and statistical analyses have been compiled by Sence Research AB. All statistical analyses were carried out using R (version 3.6.0).

## Results

In total, 1,452 patients were included in the study with a first-time diagnosis of CCS (415 women and 1,037 men). Median age was 70 years (IQR: 63–76) for women and 68 years (IQR: 62–74) for men. Median follow-up time was 3.2 (IQR: 1.4–5.8) years. During follow-up, 7.4% (*n* = 108) of patients died, and 22% (*n* = 321) experienced an ACS (Supplementary material, Figure S2). At the index CCS diagnosis, 70.4% (*n* = 292) of women and 79.2% (*n* = 821) of men underwent coronary angiography, and 31.3% (*n* = 130) of women and 44.3% (*n* = 459) of men were treated with PCI. Baseline characteristics for women and men are described in Table 1.

**Table 1:** Baseline characteristics at index CCS diagnosis.

*n*		Women	Men
		415	1,037
Age (years)	Median (IQR)	70.0 (63.0–76.0)	68.0 (62.0–75.0)
Body Mass Index (kg/m^2^)	Median (IQR)	27.9 (24.4–30.6)	27.8 (25.2–29.3)
eGFR (mL/min/1.73 m^2^)	Median (IQR)	68.9 (57.8–78.6)	69.2 (59.5–77.9)
Hypertension	*N* (%)	393 (94.7%)	953 (91.9%)
Diabetes mellitus	*N* (%)	107 (25.8%)	286 (27.6%)
Ischemic stroke	*N* (%)	15 (3.6%)	24 (2.3%)
Peripheral artery disease	*N* (%)	33 (8.0%)	93 (9.0%)
Cancer	*N* (%)	24 (5.8%)	102 (9.8%)
Charlson Comorbidity Index	Median (IQR)	1.0 (0.0–3.0)	1.0 (0.0–2.0)
Number of outpatient visits*	Median (IQR)	22.0 (13–40)	19.0 (10–32)
Medication dispensed in sachets	*N* (%)	12 (2.9%)	14 (1.4%)

*n*: number; IQR: interquartile range; eGFR: estimated glomerular filtration rate; CCS: chronic coronary syndrome.

* Within 1 year prior to the index date.

### Frequency of assessments and target achievement for blood pressure (BP)

**Figure 1:**
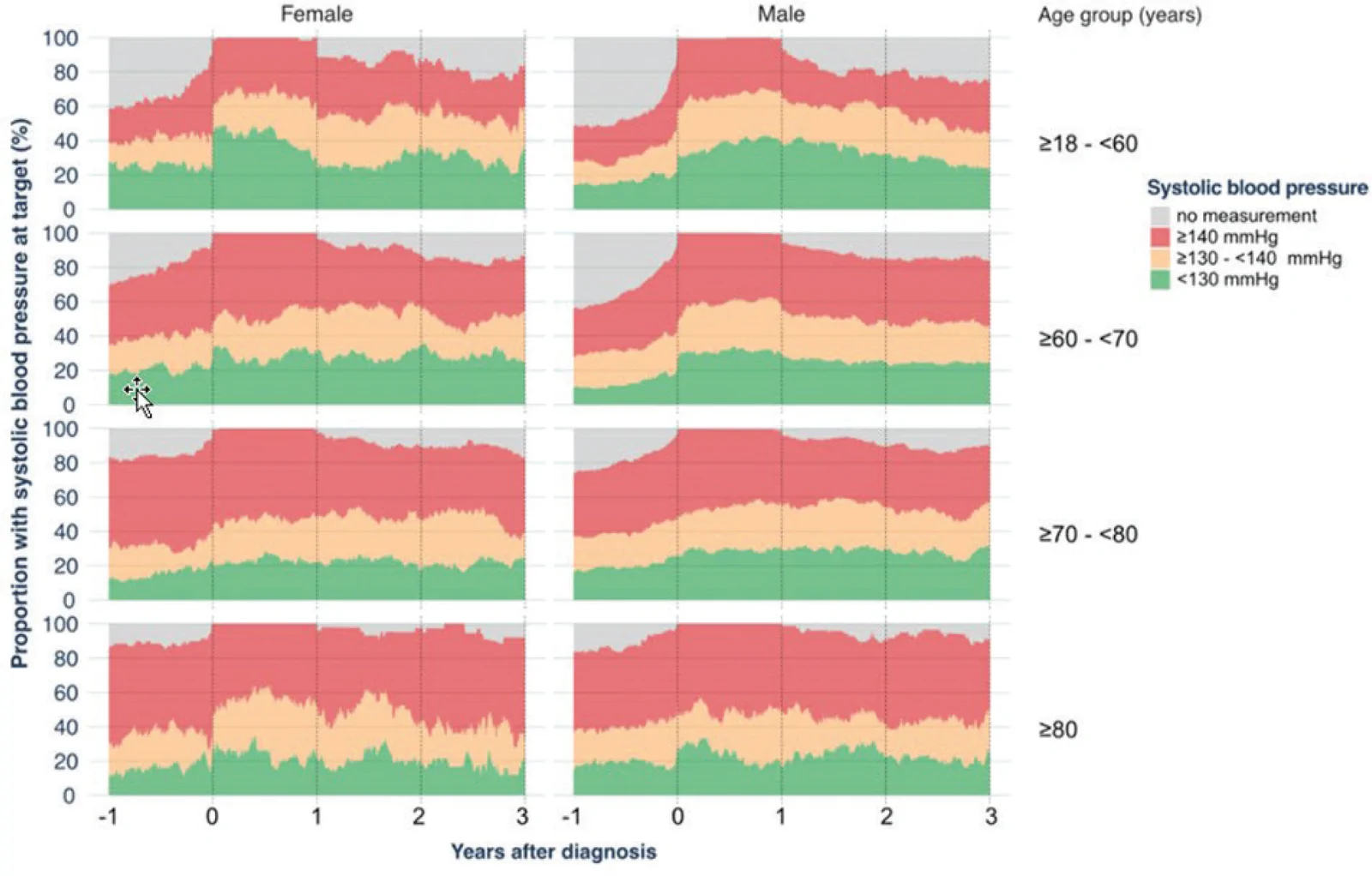
Systolic blood pressure measurements and target achievement prior to diagnosis of chronic coronary syndrome and during 3 years follow-up, by sex and age category.

One year after the index diagnosis, 4.9% of patients had no recorded BP measurement, increasing to 15.1% by the third year (Table 2, *P* < 0.001). Frequency of BP measurements was similar for women and men (year 1: *P* = 0.88, year 2: *P* = 0.29, year 3: *P* = 0.83). Patients with diabetes had more assessments during follow-up, with 4.3% missing measurements at 3 years versus 18.5% in those without diabetes (*P* < 0.001, Supplementary material, Table S2). At 1 year, 50.9% of women and 56.1% of men achieved the BP target (*P* = 0.12). Overall, 48.5% reached the systolic BP target at 3 years. Target achievement was higher among patients with diabetes, where 57.1% reached systolic BP target at 3 years versus 45.8% in those without (*P* = 0.010). Detailed target achievement by sex and age category is shown in Figure 1.

**Table 2:** Frequency of systolic blood pressure and LDL-C assessments and target achievement in patients with CCS, overall and stratified by sex.

Follow-up year	1			2			3			*P*
	Total	Women	Men	Total	Women	Men	Total	Women	Men	
*n* patients	1,159	346	813	958	296	662	756	242	514	
Systolic blood pressure (mm Hg)										
≥ 140	470 (40.6)	154 (44.5)	316 (38.9)	342 (35.7)	115 (38.9)	227 (34.3)	275 (36.4)	93 (38.4)	182 (35.4)	
130 – < 140	310 (26.7)	91 (26.3)	219 (26.9)	232 (24.2)	71 (24.0)	161 (24.3)	168 (22.2)	50 (20.7)	118 (23.0)	
< 130	322 (27.8)	85 (24.6)	237 (29.2)	259 (27.0)	77 (26.0)	182 (27.5)	199 (26.3)	64 (26.4)	135 (26.3)	
Reached target < 140	632 (54.5)	176 (50.9)	456 (56.1)	491 (51.3)	148 (50.0)	343 (51.8)	367 (48.5)	114 (47.1)	253 (49.2)	
No measurement	57 (4.9)	16 (4.6)	41 (5.0)	125 (13.0)	33 (11.1)	92 (13.9)	114 (15.1)	35 (14.5)	79 (15.4)	< 0.001
LDL-C (mmol/L)										
≥ 1.8	542 (46.8)	163 (47.1)	379 (46.6)	403 (42.1)	139 (47.0)	264 (39.9)	281 (37.2)	101 (41.7)	180 (35.0)	
1.4–< 1.8	172 (14.8)	32 (9.2)	140 (17.2)	91 (9.5)	19 (6.4)	72 (10.9)	67 (8.9)	20 (8.3)	47 (9.1)	
< 1.4	71 (6.1)	10 (2.9)	61 (7.5)	38 (4.0)	≤ 5	35 (5.3)	37 (4.9)	7 (2.9)	30 (5.8)	
Reached target < 1.8	243 (21.0)	42 (12.1)	201 (24.7)	129 (13.5)	22 (7.4)	107 (16.2)	104 (13.8)	27 (11.2)	77 (15.0)	
No measurement	374 (32.3)	141 (40.8)	233 (28.7)	426 (44.5)	135 (45.6)	291 (44.0)	371 (49.1)	114 (47.1)	257 (50.0)	< 0.001

CCS: chronic coronary syndrome; LDL-C: low-density lipoprotein-cholesterol.

Data are presented as counts (*n*) and percentages (%). The table is restricted to women and men who had complete follow-up data at 1, 2, and 3 years.

The last recorded value of the year is used to assess target achievement, and the denominator is all patients who can be followed for 1, 2, or 3 years, that is, until the end of the evaluated year.

P-values for year 1 versus year 3 comparisons were calculated using the McNemar’s test, restricted to patients with complete follow-up at both years 1 and 3 (*n* = 756).

### Frequency of assessment and target achievement for LDL-C

**Figure 2:**
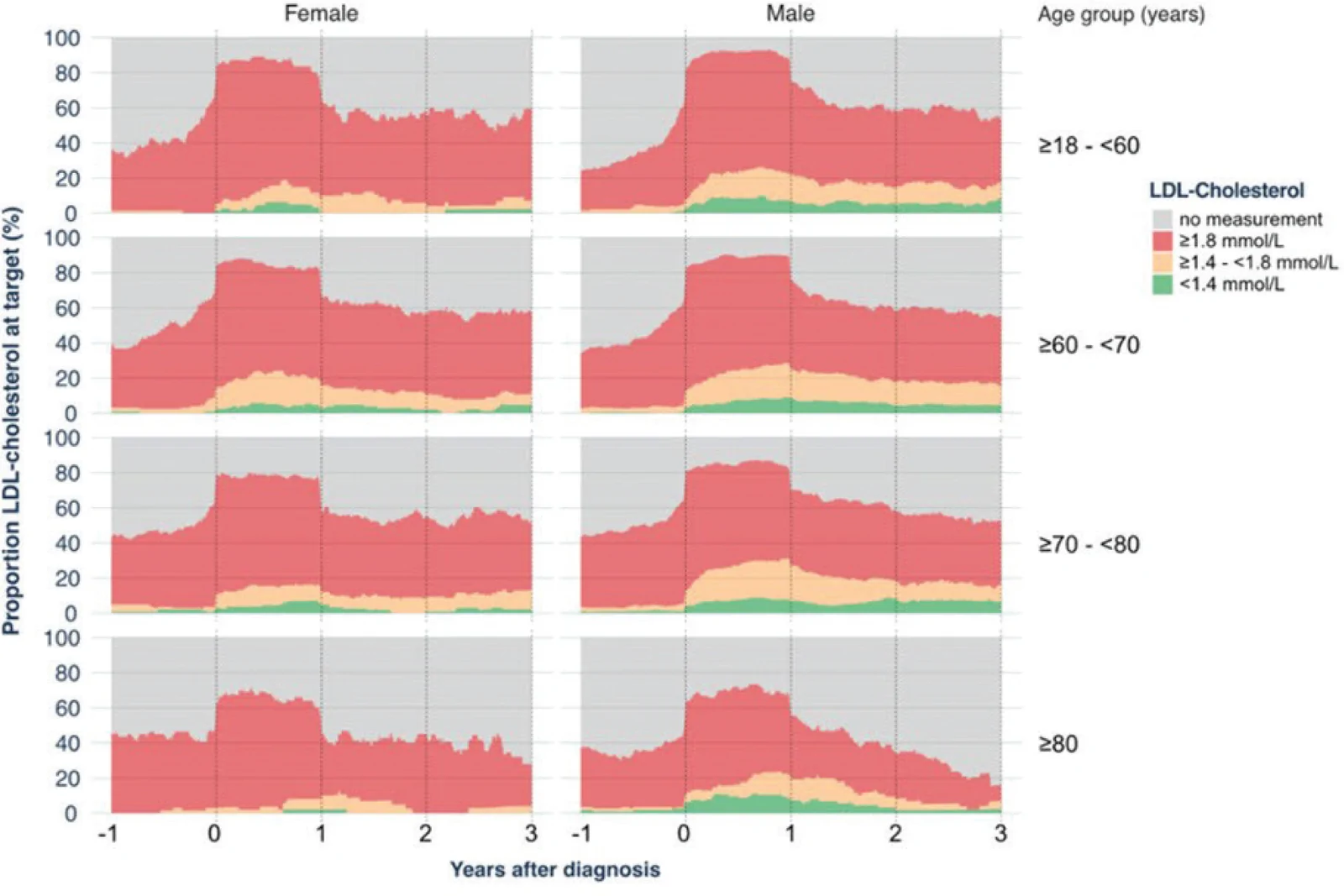
LDL-cholesterol assessment and target achievement prior to diagnosis of chronic coronary syndrome and during 3 years follow-up, by sex and age category. LDL: low-density lipoprotein.

Assessment of LDL-C declined over time with 32.3% of patients having no LDL-C measurement at 1 year, which is increasing to 49.1% by year 3 (Table 2, *P* < 0.001). Women were less likely to have an LDL-C measurement at 1 year, with 40.8% having no measurements compared to 28.7% of men (*P* < 0.001). By year 3, these numbers were more similar between women and men (47.1% vs. 50.0%, *P* = 0.51). LDL-C testing was more frequent among patients with diabetes (Supplementary material, Table S2). Across all age groups, LDL-C target achievement was lower in women than in men. At 1 year, only 12.1% of women had LDL-C levels below 1.8 mmol/L compared to 24.7% of men (*P* < 0.001). At year 3, the corresponding figures were 11.2% in women and 15.0% in men (Table 2, *P* < 0.19). Patients with diabetes showed numerically better LDL-C control overall (Supplementary material, Table S2). Detailed frequency of assessment and target achievement of LDL-C by sex and age category are described in Figure 2. Baseline characteristics of those with no LDL measurement during 1, 2, and 3 years of follow-up are described in the supplementary material (Table S3). In those with no LDL measurement, the frequency of diabetes declined over time, consistent with more structured follow-up in this group.

### Lipid-lowering therapy

**Figure 3:**
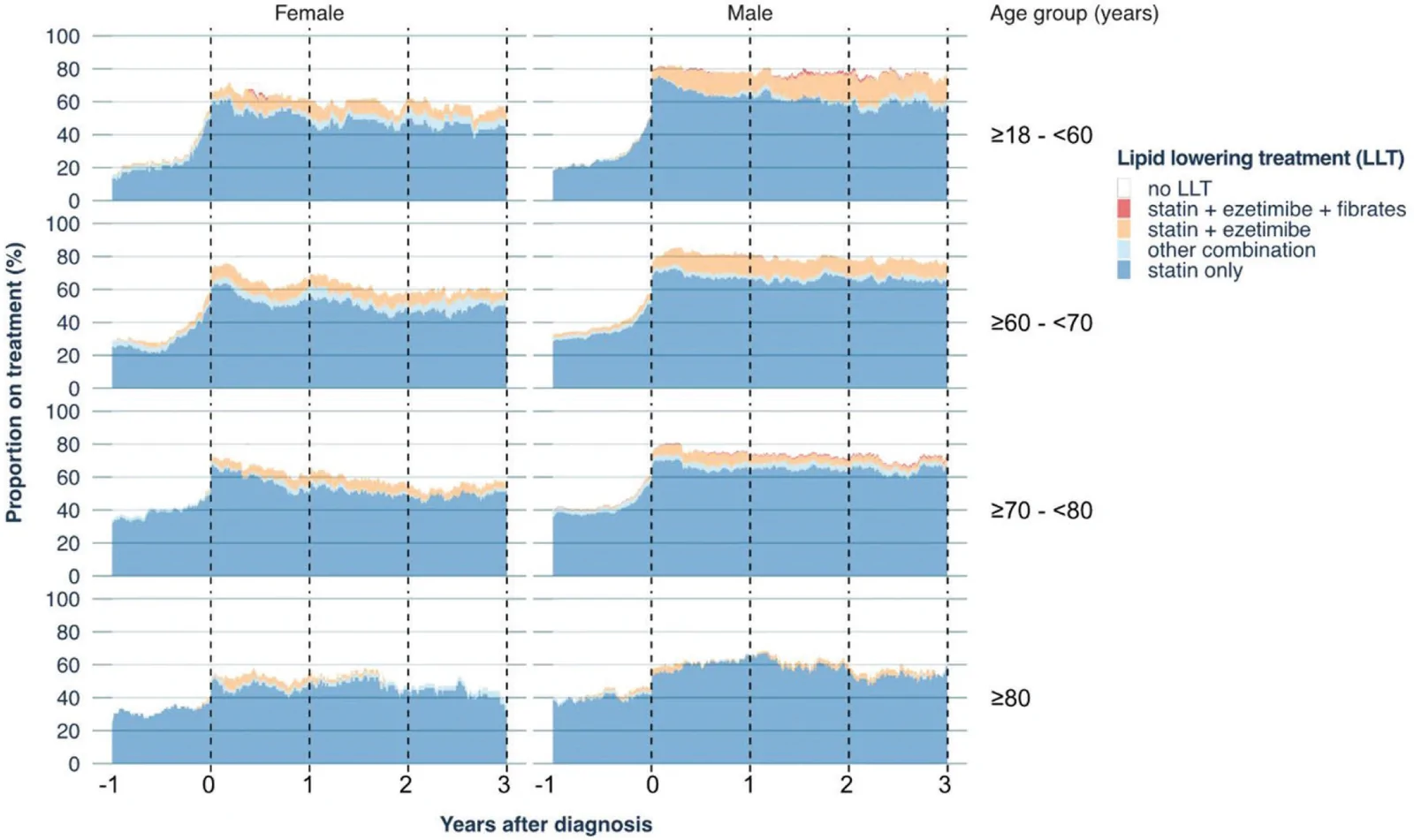
Proportion of patients on lipid-lowering therapy by sex and age category prior to chronic coronary syndrome diagnosis during 3 years follow-up.

Treatment with LLT was highest around diagnosis and declined over the following 3 years (Figure 3). No major differences in baseline characteristics were found among patients with no observed dispensation of LLT treatment within 1 year post-index as compared to those with dispensation (Supplementary material, Table S4). At year 3, 54.1% of women and 73.9% of men were on LLT (*P* < 0.001). Combination therapy with statin plus ezetimibe was less frequent among women (4.5%) than men (7.6%) (Supplementary material, Figure S3). The decline in the use of LLT was more pronounced in women than men, with 65.3% of women and 79.5% of men under 60 years of age treated with LLT at index, compared with 52.4% of women and 77.8% of men 3 years later. Furthermore, women were 30% less likely to initiate LLT within 1 year after a CCS diagnosis compared to men (adjusted hazard ratio [HR] 0.70; 95% CI: 0.61–0.80; *P* < 0.001). The corresponding E-values were 2.21 for the adjusted hazard ratio and 1.81 for the upper limit of the 95% confidence interval. Detailed information on the proportion of patients on LLT, by sex and age categories, is shown in Figure 3. Factors associated with pharmacy dispensation of LLT within 1 year after index diagnosis are described in the Supplementary material, Figure S4. The adjusted odds ratio for women receiving LLT within 1 year was 0.47 (95% CI: 0.32–0.70, *P* < 0.001).

## Discussion

This population-based study of management over time of patients with CCS describes real world data on monitoring SBP and LDL-C, which is not captured in the national quality registers, including sex differences in treatment patterns. There were four key findings of the study. First, despite repeated healthcare contacts measuring blood pressure, the frequency of LDL-C assessments was low and decreased over time. Second, the proportion of individuals achieving LDL-C targets was low and declined every year reaching ~10–15% at 3 years. Third, despite detection of blood pressure and LDL-C being at levels associated with harm, limited mitigation seemed to occur with consistent elevated levels over time. Fourth, women had lower frequency of LDL-C assessments and lower target achievement than men and were less often treated with LLT, regardless of age, with a 30% lower hazard of initiating LLT within 1 year after a CCS diagnosis compared to men.

In this study, the frequency of blood pressure assessments was markedly higher than for LDL-C assessments. For patients in primary care following a national structured scheme, such as those with diabetes, the frequency of LDL-C measurements was higher and, in many cases, occurred annually. In Sweden, there is a general lack of physicians specialized in general medicine often with poor continuity in care as a result. Continuity of care has previously been associated with increased statin adherence in a primary care setting (18). The recommended frequency of monitoring lipids in secondary prevention has been suggested to 3–12 months according to a systematic review of several international clinical guidelines (19). Our study shows that most CCS patients followed in primary care, in a region with demographic characteristics representative of Sweden, do not have annual assessments of lipids. Patients with diabetes (most of them with type 2 diabetes) attaining the standardized follow-up in primary care with regularly visits to a specialized nurse had a higher frequency of LDL-C assessments. The standardized format of care has been shown to contribute to a higher target achievement in this population (20). Patients with CCS but without diabetes could potentially benefit from a standardized follow-up in primary care, similar to the one used for patients with diabetes.

The results of our study are in line with those of EUROASPIRE V, with better LDL-C control in men than in women, and in patients with diabetes compared to those without (4). A Swedish register-based study assessing blood pressure and LDL-C management in patients after ACS also showed similar results. In this study, the patients were followed in primary care during 5 years with low adherence to treatment and also low control of both blood pressure and LDL-C over time. Furthermore, patients with worse LDL-C control had a higher risk of recurrent ACS and death (21). Several international register-based studies show suboptimal control of LDL-C in patients at high risk or very high risk of CVD, with the vast majority being far from prevention target achievement (22, 23). It has been proposed that there is a need of more modern tools in optimizing secondary prevention treatments (24), such as clinical decision support systems albeit with a variability in approaches and heterogeneity in results after implementation (25).

A study of CCS patients found that women were older than men, with a worse cardiovascular risk factor profile and lower rates of coronary revascularization (26). The CLARIFY study, an international multi-center study including patients with CCS, found similar 5-year event rates in women and men (27). Despite this, there is evidence of sex differences in the use of LLT at all stages, such as counseling, prescription, adherence, and monitoring (28). Age has been suggested as an explanatory factor, but in this study, we found lower frequency of LLT both in younger and older women. Patient-reported statin intolerance and side effects have also been proposed as an explanatory factor accounting for the lower prescription in women but could not be studied further in this study. Statin intolerance and side effects occur in an important minority of patients, with increased risks of outcome in women, at older age and at higher doses (29).

The Swedish Association of General Practice has recently advised against repeated lipid measurements in patients with a well-functioning treatment, as a part of their choosing wisely initiative (30). The arguments against repeated lipid testing stem from a critique of standardized care, although it is recognized that standardization can improve patient safety in understaffed centers. Key reasons for questioning repeated lipid testing and the treat-to-target approach include time constraints (31, 32), which limit the availability for preventive care, and the variability between test results due to analytical and biological factors (33). Similar to our study, it has been shown that repeated testing rarely alters the treatment strategy, particularly in patients in primary prevention (34). However, the latter study did not assess if patients were at treatment targets or not, a key factor in refraining from optimizing risk reducing therapies. Moreover, the Association for General Practice does not address the differences between primary and secondary prevention strategies. The ‘fire and forget’ strategy of using the highest tolerated dose of statin instead of the traditional treat-to-target approach has been suggested as more cost-effective (35). However, the former strategy is only working if an adequate intensity in treatment is prescribed. Our study concludes that many patients remain undertreated, and women were less likely than men to receive LLT, either in monotherapy or as combination with statin and ezetimibe. In a study of Bentzel et al, repeated monitoring of LDL-C was associated with a more favorable outcome (21). To determine the highest tolerated dose in each patient, there is a need of a structured follow-up of eventual side effects of LLT, which, in some cases, need to be evaluated by titration of the dose. The effectiveness of LLT must be evaluated through repeated LDL-C measurements. If treatment response is inadequate, the responsible physician should assess potential causes in a patient-centered manner, including repeat laboratory assessments of LDL-C. In our study, where treatment targets were achieved in only one in five patients, it cannot be elucidated that therapy was effective, especially given that women were undertreated.

### Strengths and limitations

The Uppsala Region includes both the fourth largest city of Sweden and large areas in the countryside with the same EHRs system both in the hospital and in the majority of the primary care centers. Patients were included in the study without restrictions, and the study reflects clinical routine care with its limitations. Since the study was observational, there is a risk of residual confounding. The adjustment for confounding has been described in detail. An impact of unmeasured confounding could not be ruled out, and the study lacked information on socioeconomics, adherence to medical treatment and treatment intensity. Although caregiver inertia, caregiver prescribing willingness, and LLT persistence are important determinants of lipid target achievement, these could not be formally assessed in the present study and warrant dedicated investigation in future research. The quality of the registrations in the EHR of each responsible physician could have an impact on the results with a potential risk of misclassification bias. In office, SBP measurements were used, due to a limitation in registration of home-registered SBP in the EHR.

As in any observational study, unmeasured confounding cannot be excluded. The E-value for the adjusted hazard ratio was 2.21, and 1.81 for the upper limit of the 95% confidence interval. To fully explain the observed sex difference, an unmeasured confounder would need to be associated with both female sex and LLT initiation by a risk ratio of at least 2.21, not accounted for by the measured covariates; an association of at least 1.81 would be required to shift the confidence interval to include the null. Confounding of this strength is unlikely to fully account for the observed difference.

## Conclusions

Only a minority of patients achieved recommended LDL-C targets, and approximately half achieved blood pressure targets. The use of LLT declined markedly over time. Women less often received LDL-C monitoring and LLT than men, while patients with diabetes, enrolled in a standardized follow-up scheme, showed better control. Our findings reveal large gaps in secondary prevention, highlighting the need for systematic, guidelines-based management of patients with CCS in primary care.

### Ethics statement

This study was approved by the Swedish Ethical Review Authority (reference number 2019-06295). This study was conducted according to the declaration of Helsinki and institutional guidelines. The requirement of informed consent was waived by the institutional review board.

## Supplementary Material



## Data Availability

Data are available upon reasonable request.
