# Supplementary material for: Sex differences in monitoring and achievement of blood pressure and LDL-cholesterol targets in chronic coronary syndrome
Source: Ups J Med Sci. 2026 Aug 31;131:10.48101/ujms.v131.14047. doi: 10.48101/ujms.v131.14047 (PMC13563433; doi:10.48101/ujms.v131.14047)
Supplement: Supplementary file 1 [file UJMS-131-14047-s1.pdf]

Supplemental material

Figure legends

Figure S1: Number of CCS events distributed over the municipalities of Region Uppsala, Sweden

The colour intensity is proportional to the number of events.

The cardiology clinics of Region Uppsala are shown in orange

UAS, Uppsala University Hospital; CCS, Chronic coronary syndrome

Distribution of study participants described in percent

Figure S2: Loss of Follow-up over time for CCS

ACS, acute coronary syndrome

Figure S3: Proportion of patients on lipid-lowering therapy by sex prior to CCS diagnosis during three years follow-up

CCS, chronic coronary syndrome

Figure S4: Factors for pharmacy dispensation of lipid-lowering therapy within one year after first CCS event

On X axis is Odds ratio (standard error, p-value)

Results from a multivariable logistic regression model. All listed variables were included simultaneously as independent variables. The dependent variable was LLT dispensation within one year after index. On X axis: odds ratio (standard error, p-value).

*= within one year prior to index date

BMI, body mass index; eGFR, estimated glomerular filtration rate; PCI, percutaneous coronary intervention; LLT, lipid lowering therapy.

List of tables

Table S1: ICD and procedure codes

Table S2: Frequency of systolic blood pressure and LDL-C assessments and target achievement in patients with CCS, stratified by diabetes mellitus

Table S3: Baseline characteristics of patients with no LDL monitoring during 1, 2 and 3 years of follow-up

Table S4: Baseline characteristics at index (first CCS event) among those patients with no observed pharmacy dispensation of lipid lowering therapy within one-year post-index

Figure S1: Number of CCS events distributed over the municipalities of Region Uppsala, Sweden

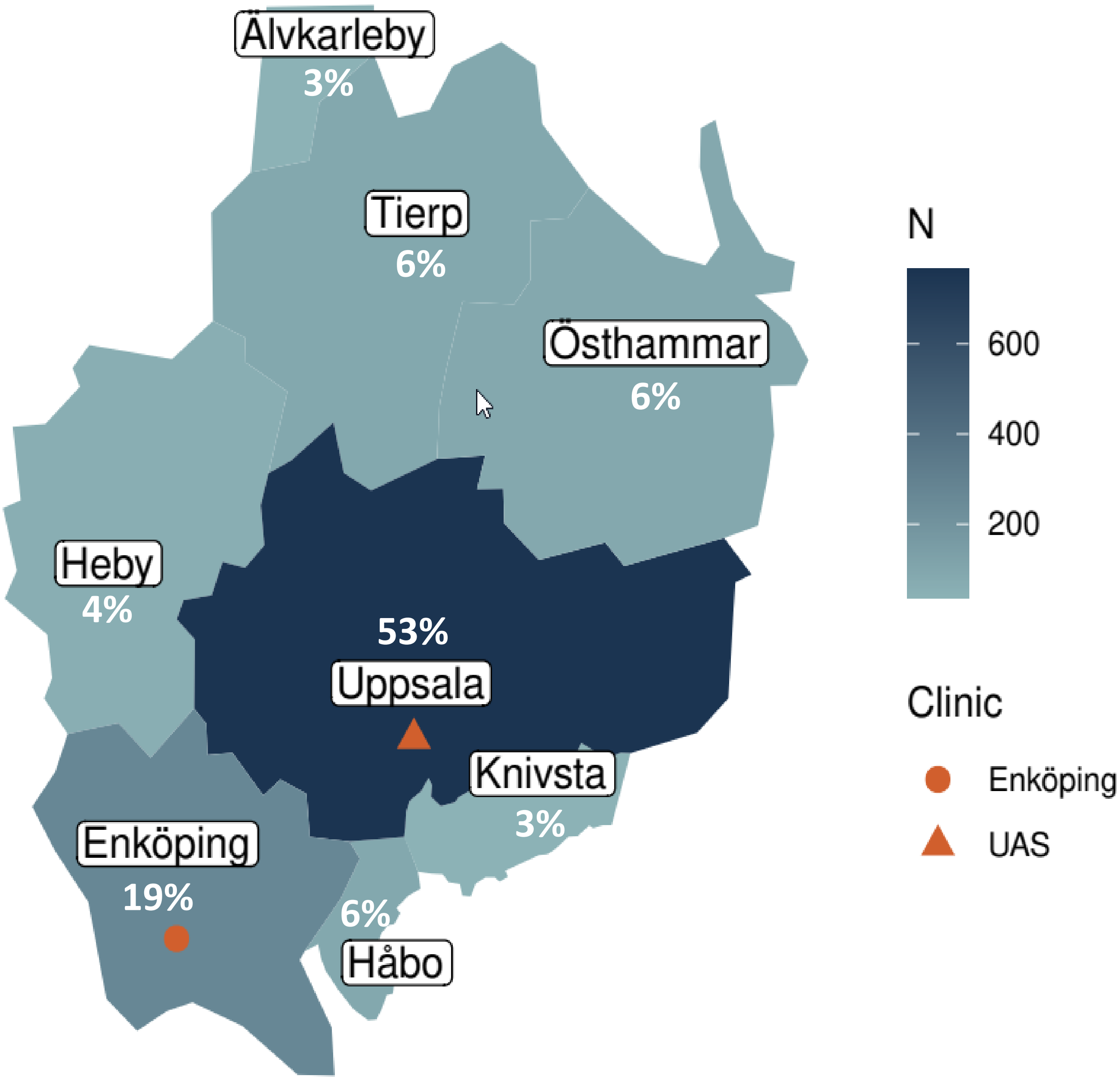

Figure S2: Loss of follow-up after study index

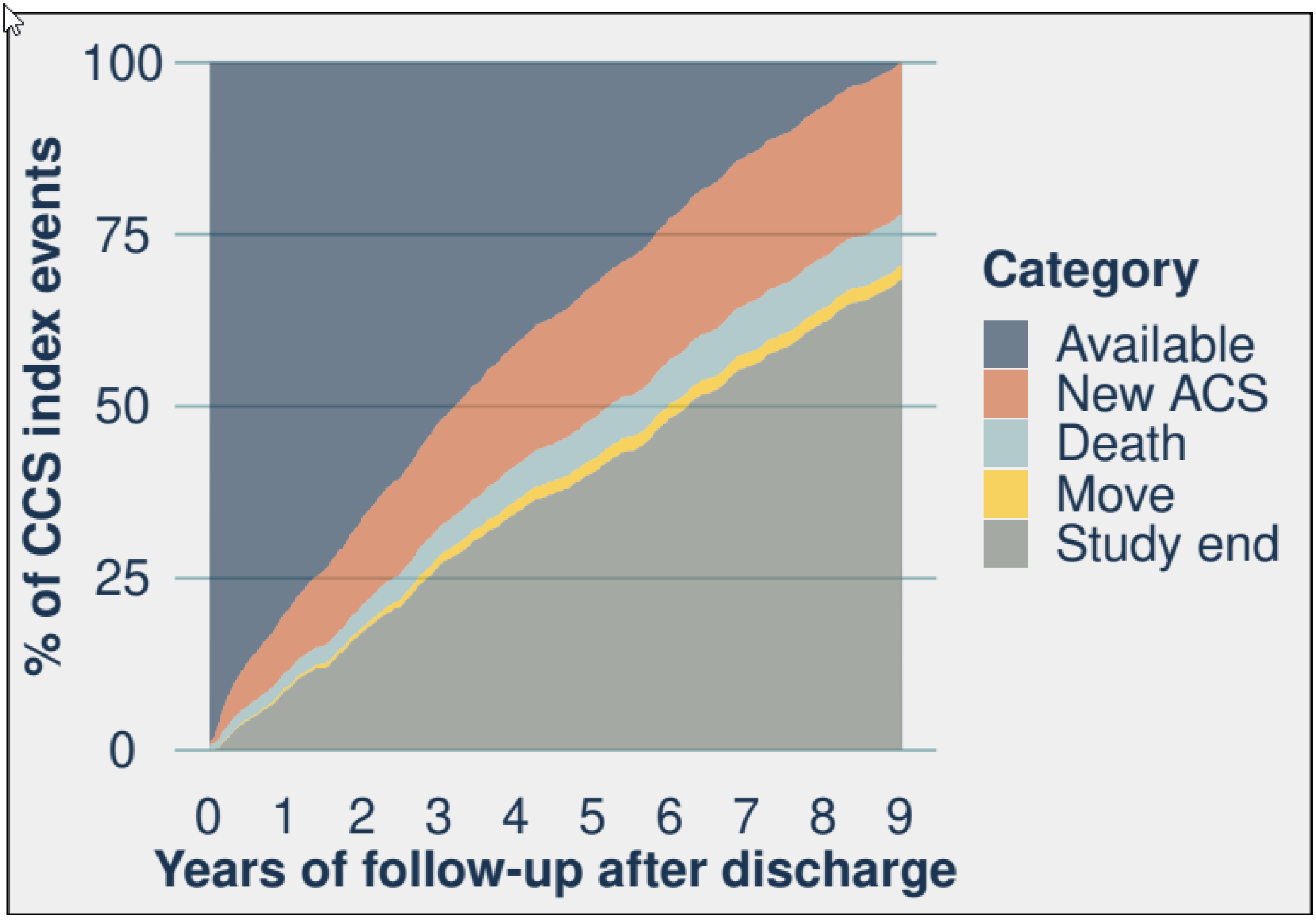

Confidential Medical and Scientific Affairs

Figure S3: Proportion of patients on lipid-lowering therapy by sex prior to CCS diagnosis during three years follow-up

A. Women

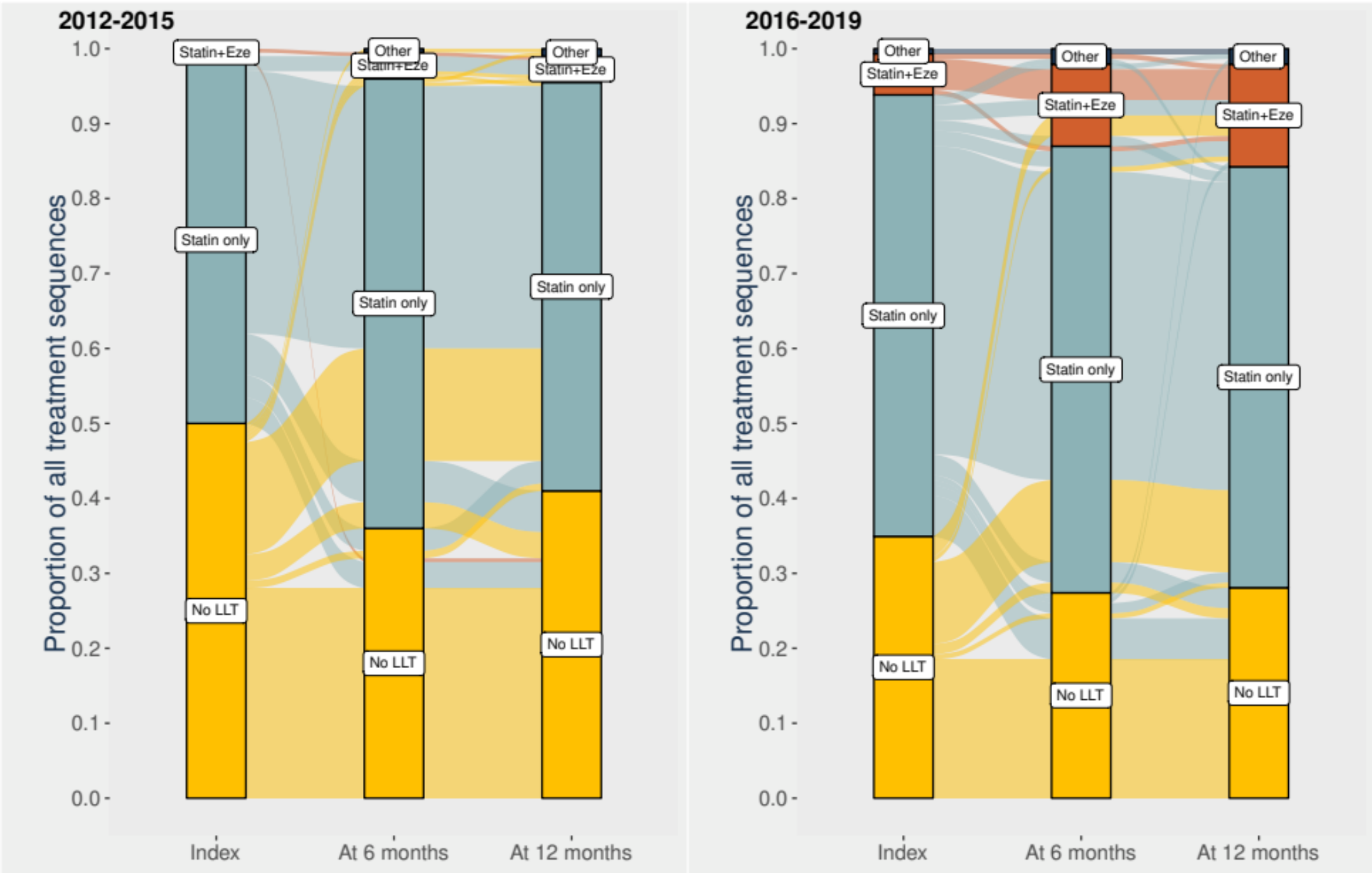

B. Men

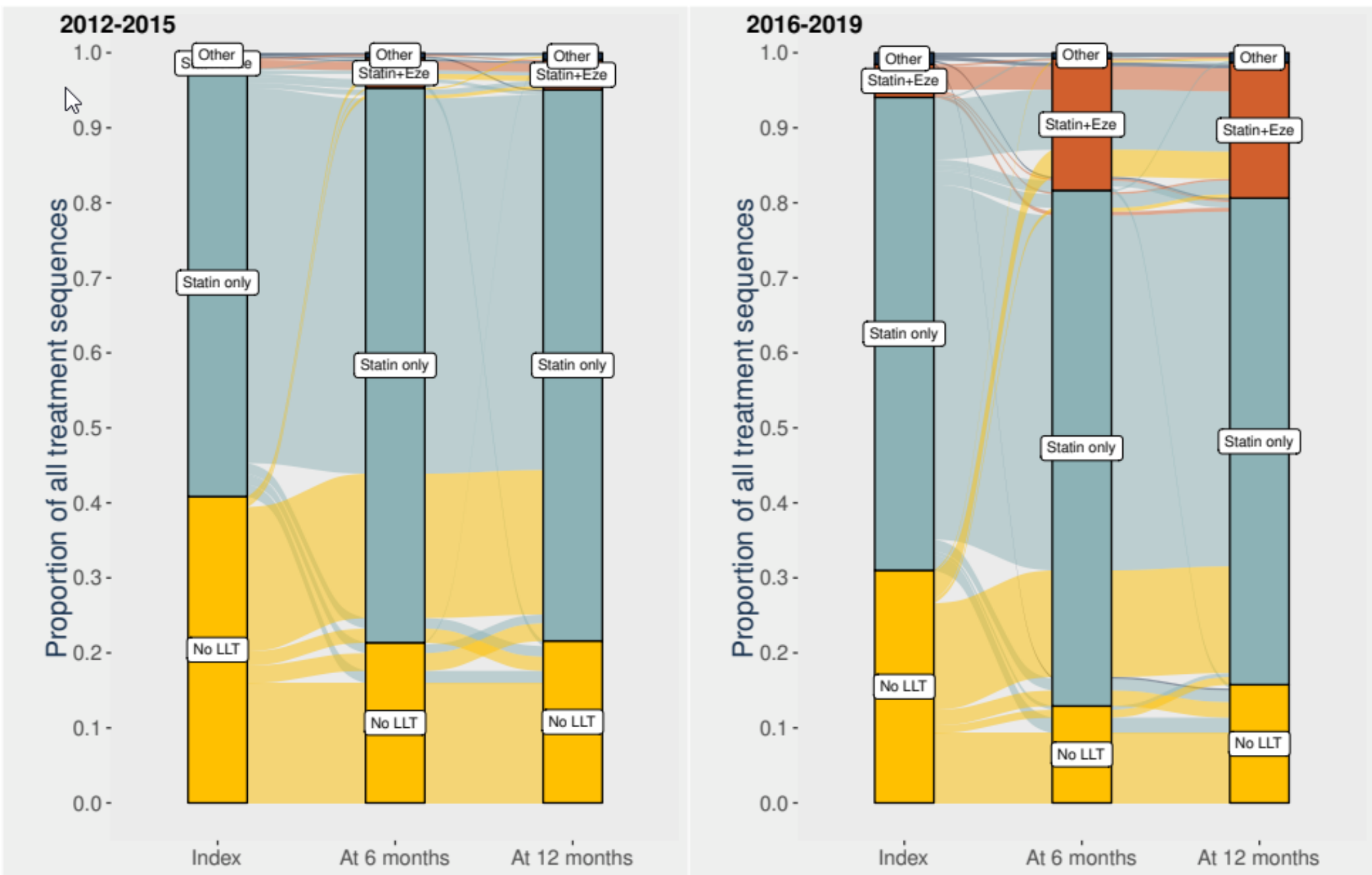

Confidential Medical and Scientific Affairs

Figure S4: Factors associated with pharmacy dispensation of lipid lowering therapy within one year after index diagnosis

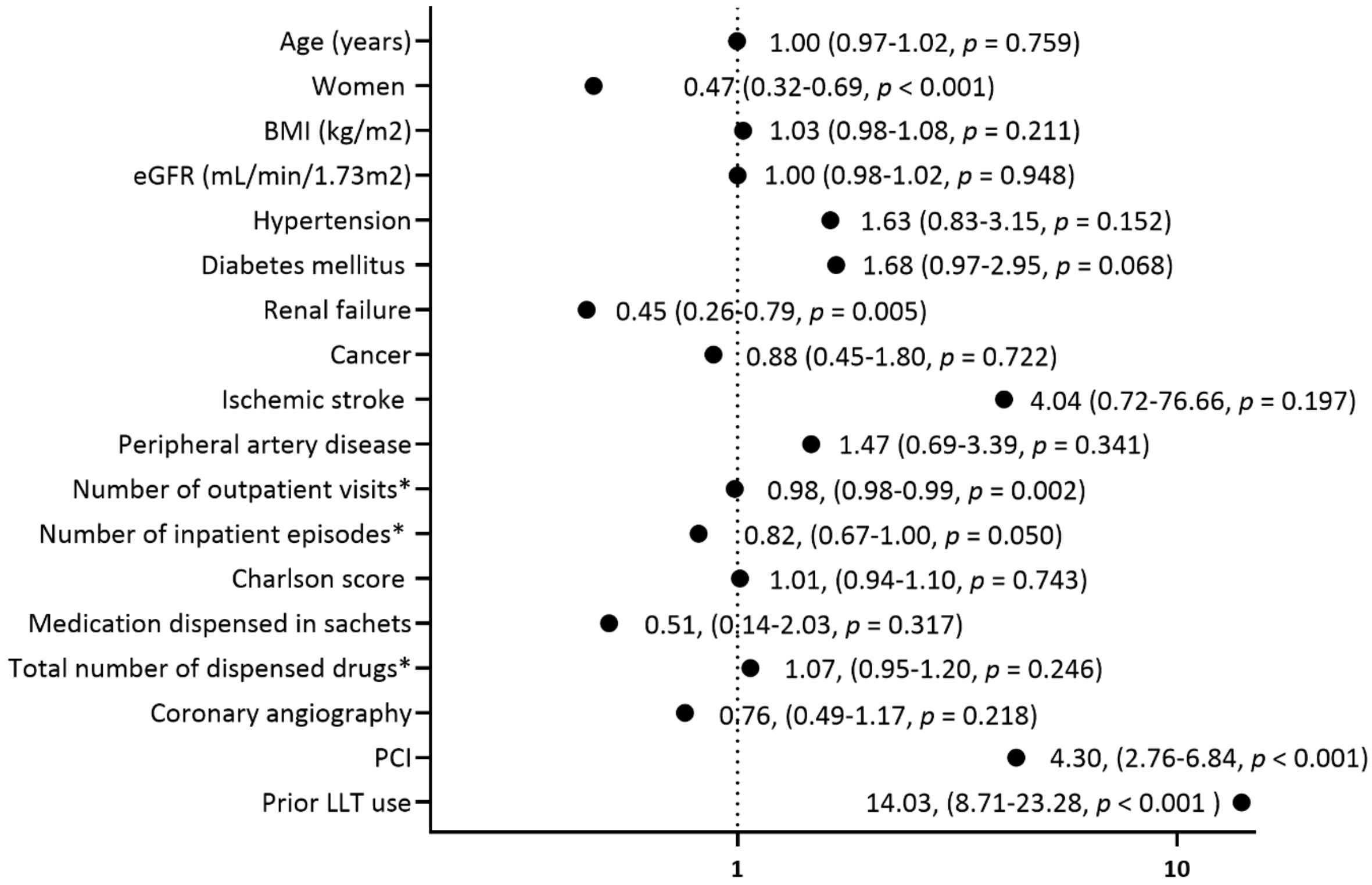

Results from a multivariable logistic regression model. All listed variables were included simultaneously as independent variables. The dependent variable was LLT dispensation within one year after index. On X axis: odds ratio (confidence interval, p-value).

*= within one year prior to index date

BMI, body mass index; eGFR, estimated glomerular filtration rate; PCI, percutaneous coronary intervention; LLT, lipid lowering therapy.

Table S1: ICD and procedure codes

Diagnosis	Definitions	ICD-10 code
**Chronic coronary syndrome**	Described in the manuscript	I20.[8, 9] or I25.[0, 1, 5, 6, 8, 9]
**Comorbidities**		
**Diabetes mellitus**	HbA1c > 48 mmol/L or prescription of blood glucose–lowering treatment	E10-E14
**Dyslipidaemia**	LDL-C > 3.0 mmol/L, triglycerides > 2.0 mmol/L, total cholesterol > 4.0 mmol/L or LLT	E78
**Hypertension (primary)**	SBP ≥ 140 mmHg<br>DBP ≥ 90 mmHg<br>dispensation of antihypertensive medication*	I10.9
**Ischemic stroke**	-	I63
**PAD**	-	I70-I72, I73.1, I73.9, I74, I77.3, I77.6, I77.8, I79
**Chronic kidney failure**	eGFR < 60 mL/min/1.73 m² or<br>a urine albumin–creatinine ratio > 3 mg/mmol	N17-N19
**Cancer**	-	C (excluding C44, basal cell carcinoma)

Procedures		Procedures codes
**Coronary angiography**	-	AF037
**Invasive coronary intervention**	-	FNG
**Non-invasive coronary intervention**	-	AF032, AF060, AV032, AF012, AF018, AF019, AF045, AF046, AF054, AF062, AF061, AV063, AV064, AV065

All medical treatment were considered within one year before the index date.
LLT, lipid lowering therapy; SBP, systolic blood pressure; DBP, diastolic blood pressure

* = alpha-blocker, beta-blocker, calcium channel blocker, or angiotensin-converting enzyme inhibitor or angiotensin receptor blocker

Table S2: Frequency of systolic blood pressure and LDL-C assessments and target achievement in patients with CCS, stratified by diabetes mellitus

Follow-up year	1		2		3		Group comparison year 3 (p-value)
	DM	No DM	DM	No DM	DM	No DM	
**n events**	303	856	241	717	184	572	
**Systolic blood pressure (mmHg)**							
≥ 140	116 (38.3)	354 (41.4)	90 (37.3)	252 (35.1)	71 (38.6)	204 (35.7)	
130 - < 140	95 (31.4)	215 (25.1)	62 (25.7)	170 (23.7)	45 (24.5)	123 (21.5)	
< 130	84 (27.7)	238 (27.8)	76 (31.5)	183 (25.5)	60 (32.6)	139 (24.3)	
Reached target <140)	179 (59.1)	453 (52.9)	138 (57.3)	353 (49.2)	105 (57.1)	262 (45.8)	0.010
**No measurement**	8 (2.6)	49 (5.7)	13 (5.4)	112 (15.6)	8 (4.3)	106 (18.5)	<0.001
**LDL-C (mmol/L)**							
≥ 1.8	156 (51.5)	386 (45.1)	132 (54.8)	271 (37.8)	87 (47.3)	194 (33.9)	
1.4 - < 1.8	49 (16.2)	123 (14.4)	40 (16.6)	51 (7.1)	35 (19.0)	32 (5.6)	
< 1.4	23 (7.6)	48 (5.6)	16 (6.6)	22 (3.1)	13 (7.1)	24 (4.2)	
Reached target < 1.8	72 (23.8)	171 (20.0)	56 (23.2)	73 (10.2)	48 (26.1)	56 (9.8)	<0.001
**No measurement**	75 (24.8)	299 (34.9)	53 (22.0)	373 (52.0)	49 (26.6)	322 (56.3)	<0.001

Data are presented as counts (n) and percentages (%). The table is restricted to those who had complete follow-up data at 1, 2, and 3 years, respectively.

DM, diabetes mellitus; LDL-C, low-density lipoprotein-cholesterol

Table S3: Baseline characteristics of patients with no LDL monitoring during 1, 2 and 3 years of follow-up

		**1 year**	**2 years**	**3 years**
**No LDL measurement**	n	374	209	126
**Total**	n	1159	958	756
**Age**	Median (IQR)	70 (63-78)	70 (63-78)	70 (62-78)
**Women**	n (%)	141 (37.7%)	81 (38.8%)	43 (34.1%)
**Body mass index (kg/m^2)**	Median (IQR)	27.7 (24.9-29.2)	27.2 (24.3-29.1)	26.8 (24.3-28.7)
**eGFR ($mL/min/1.73m^2$)**	Median (IQR)	68.3 (58.6-77.1)	67.3 (59.2-76.7)	67.2 (58.9-79.7)
**Hypertension**	n (%)	345 (92.2%)	192 (91.9%)	113 (89.7%)
**Diabetes mellitus**	n (%)	75 (20.1%)	25 (12.0%)	11 (8.7%)
**Ischemic stroke**	n (%)	13 (3.5%)	6 (2.9%)	<5
**Peripheral artery disease**	n (%)	28 (7.5%)	17 (8.1%)	6 (4.8%)
**Cancer**	n (%)	30 (8.0%)	18 (8.6%)	11 (8.7%)
**Charlson Comorbidity Index**	Median (IQR)	1.0 (0.0-2.0)	1.0 (0.0-2.0)	1.0 (0.0-2.0)
**Number of outpatient visits**	Median (IQR)	19.0 (10.0-35.0)	19.0 (9.0-38.0)	17.0 (9.0-31.5)
**Previous LLT**	n (%)	200 (53.5%)	101 (48.3%)	56 (44.4%)

eGFR, estimated glomerular filtration rate; LLT, lipid lowering therapy

Confidential Medical and Scientific Affairs

Table S4: Baseline characteristics at index (first CCS event) among those patients with no observed pharmacy dispensation of lipid lowering therapy treatment within one-year post-index

		**Women**	**Men**
**N**		95	115
**Age**	Median (IQR)	70.0 (64.0-78.5)	71.0 (62.0-78.0)
**Body mass index (kg/m^2)**	Median (IQR)	27.3 (23.6-30.5)	26.6 (24.6-28.6)
**eGFR ($mL/min/1.73m^2$)**	Median (IQR)	70.1 (56.2-78.9)	67.5 (55.8-76.4)
**Hypertension**	n (%)	86 (90.5%)	101 (87.8%)
**Diabetes mellitus**	n (%)	14 (14.7%)	18 (15.7%)
**Ischemic stroke**	n (%)	≤ 5	≤ 5
**Peripheral artery disease**	n (%)	≤ 5	9 (7.8%)
**Cancer**	n (%)	7 (7.4%)	11 (9.6%)
**Charlson Comorbidity Index**	Median (IQR)	1.0 (0.0-3.0)	1.0 (0.0-2-0)
**Number of outpatient visits***	Median (IQR)	22.0 (12.5-44.0)	22.0 (12.0-40.5)
**Previous LLT**	n (%)	18 (19.0%)	13 (11.3%)

eGFR, estimated glomerular filtration rate; LLT, lipid lowering therapy

* Within one year prior to the index date. This analysis was performed as follows: patients with at least 365 days of follow-up were identified. Among these, patients with no pharmacy dispensation of LLT within one year after the index date were selected. Patients may have received LLT prior to the index CCS event.

Confidential Medical and Scientific Affairs
